# Strategies of *Phytophthora infestans* adaptation to local UV radiation conditions

**DOI:** 10.1111/eva.12722

**Published:** 2018-11-06

**Authors:** E‐Jiao Wu, Yan‐Ping Wang, Lin‐Lin Shen, Lurwanu Yahuza, Ji‐Chen Tian, Li‐Na Yang, Li‐Ping Shang, Wen Zhu, Jiasui Zhan

**Affiliations:** ^1^ State Key Laboratory of Ecological Pest Control for Fujian and Taiwan Crops Fujian Agriculture and Forestry University Fuzhou Fujian China; ^2^ Fujian Key Laboratory of Plant Virology, Institute of Plant Virology Fujian Agricultural and Forestry University Fuzhou Fujian China; ^3^ Department of Forest Mycology and Plant Pathology Swedish University of Agricultural Sciences Uppsala Sweden

**Keywords:** aggressiveness, genetic differentiation, pathogen evolution, phenotypic plasticity, *Phytophthora infestans*, *Q*_ST_/*F*_ST_ analysis, ultraviolet adaptation

## Abstract

Expected global changes in environmental conditions underline the need for a better understanding of genetic variation in ecological traits and their strategies of adaptation to the stresses. In this study, evolutionary mechanisms and processes of UV adaptation in plant pathogens were investigated by combining statistical genetics, physiological assays, and common garden experiment approaches in an assessment of the potato late blight pathogen, *Phytophthora infestans,* sampled from various geographic locations in China. We found spatial divergence caused by diversifying selection in UV tolerance among *P. infestans *populations. Local UV radiation was the driving force of selection as indicated by a positive correlation between UV tolerance in *P. infestans* populations and the altitude of collection sites. Plasticity accounted for 68% of population variation while heritability was negligible, suggesting temporary changes in gene expression and/or enzymatic activity play a more important role than permanent modification of gene structure in the evolution of UV adaptation. This adaptation strategy may explain the lack of fitness penalty observed in genotypes with higher UV tolerance.

## 1 INTRODUCTION

Natural and agricultural ecosystems play an important role in the food security, human health, and social stability (Fisher et al., 2012; Rúa, Mcculley, & Mitchell, 2014). The structure, function, and sustainability of current ecological systems in the planet are challenged by ongoing climate changes (Waters et al., 2016) believed to be mainly caused by anthropogenic activities (Gleick et al., 2010). Knowledge on the evolutionary adaptation of species to environmental conditions through modification of physiological, biochemical, and genetic characteristics is critical for coping with the ecological challenges caused by the climate changes. A key challenge in evolutionary biology is thus to understand when and how adaptive events may mitigate the impacts of changing climatic conditions and to determine what mechanisms and processes are underlying these adaptations. This information is critical for designing management programs that minimize the negative impacts of climate change on sustainability and human society (Hoffmann & Sgrò, 2011).

For an effect as potentially catastrophic as global warming (Huang et al., 2017), elevated UV radiation resulting from ozone depletion (Listed, 2011; Paul, 2000) and changing solar activity (Williamson et al., 2017) pose an ongoing threat to life on earth. UV is one of the most important environmental parameters with crucial impacts on genetic, biochemical, physiological, and ecological processes of organisms. It induces flavonoid production, alters secondary metabolism activity, enhances photo‐protection, and up‐regulates antioxidative response of cells (Bassman, 2004; Mazza, Giménez, Kantolic, & Ballaré, 2013; Wargent & Jordan, 2013). It also reduces proofreading efficiency of cells during DNA replication and therefore has a mutagenic effect (Gruijl, Kranen, & Mullenders, 2001; Pfeifer, You, & Besaratinia, 2005; Shibai et al., 2017). Tolerance to UV radiation in organisms is determined by their protective mechanisms that prevent or reduce the occurrence of damage to intracellular components, and by systems that can repair the damage caused by radiation (Braga, Rangel, Flint, Anderson, & Roberts, 2006; Chelico, Haughian, Woytowich, & Khachatourians, 2005). In plants, UV radiation reduces tissue growth (i.e., leaf area expansion) and photosynthesis (Mazza et al., 2013; Schmitzhoerner & Weissenböck, 2003). In fungi, UV radiation negatively influences their production, survival, dispersion, distribution, germination, pathogenicity, and virulence (Braga, Rangel, Flint, & Roberts, 2015; Cheng et al., 2014; Corrochano & Garre, 2010; Idnurm, Verma, & Corrochano, 2010).

In the antagonistic interaction between hosts and pathogens, UV radiation can affect disease initiative, development, and epidemics by modifying pathogen colonization, growth, reproduction, and pathogenicity, or altering the morphological structure and physiological and biochemical processes of hosts (Charles, Benhamou, & Arul, 2008; Charles, Tano, Asselin, & Arul, 2009; Christie et al., 2012; de Menezes et al., 2015). Many plant, animal, and human diseases are expected to shift in their geographic distribution and abundance in responses to alterations in the intensity of UV radiation (Altizer, Ostfeld, Johnson, Kutz, & Harvell, 2013). These effects of change in the Earth's UV radiation patterns on disease ecology are inextricably linked to human health and ecological sustainability. However, the ultimate direction and extent of these changes in disease ecology rely on population variation, evolutionary mechanisms and landscapes of pathogens, and the multifaceted interactions of pathogen species with biotic and abiotic environments such as hosts and non‐pathogenic microbes (Fitt, Huang, Bosch, & West, 2006; Häder, Kumar, Smith, & Worrest, 2007). Fisher's fundamental theorem of natural selection postulates that the adaptive ability of natural populations to changing environments depends on heritable variation in ecological and morphological traits that are relevant to fitness (Fisher, 1930). Natural variation in pathogen populations can be additive (genetic variation that contributes to the total phenotypic variation of a quantitative trait) generated and maintained by mutation through change in nucleotide sequences of genomes. Due to short generation times and large population sizes (Zhan, Thrall, & Burdon, 2014; Zhan, Thrall, Papaix, Xie, & Burdon, 2015), pathogens can quickly accumulate large numbers of mutations in a relatively short timescale relative to their hosts. However, natural variation through gene mutation may carry a fitness penalty because mutations often impede important cellular and biochemical functions of genes to adapt for particular environments (Roles, Rutter, Dworkin, Fenster, & Conner, 2016). Natural variation in pathogen populations can also be generated by plasticity through changes in gene expression and/or enzymatic activity as a response of species to environmental stresses. Plasticity, a fundamental component of fitness, which is limitless, reversible, and metabolically inexpensive (Callahan, Maughan, & Steiner, 2008; Scheiner, 1993), is critical for the rapid adaptation of species to environments well before allelic adaptation can take place (Hendry, Farrugia, & Kinnison, 2008). This is particularly important in the adaptation of species to environments experiencing constant and rapid changes such as air temperature (Yang et al., 2016).

The proximate and ultimate mechanisms and evolutionary landscape of UV adaptation in pathogens are poorly understood and often based on anecdotal and non‐synthesized information. Here, we present a combination of statistical genetics, molecular marker, and an experimental evolution approach, to understand the evolutionary processes, genetic mechanisms, and trade‐offs involved in UV adaptation in the potato late blight pathogen, *Phytophthora infestans.* We do this by contrasting patterns of genetic variation at neutral simple sequence repeat (SSR) markers and in UV tolerance and evaluating the association between UV tolerance and aggressiveness in the pathogen. *Phytophthora infestans* (Mont) de Bary is the most devastating potato pathogen worldwide (Fry, 2008), causing approximately eight billion US dollars direct economic loss annually (Haverkort et al., 2008; Runno‐Paurson et al., 2013). The pathogen can affect all parts of the potato including leaves, stems, and tubers and may destroy entire crops within a few days. However, *P. infestans* is very sensitive to UV radiation. The germination and viability of its sporangia and other reproductive units can be significantly reduced even when they are shortly exposed to low doses of UV radiation (Belmar‐Diaz et al., 2005; Mizubuti, Aylor, & Fry, 2000; Oijen, 1991).

Because of this, the specific objectives of our study were to: (a) evaluate the relative contribution of gene divergence accumulated by mutation, and plasticity generated by change in gene expression and/or enzymatic activity, to the UV adaptation of *P. infestans*; (b) determine the historical role and causal components involved in the evolution of UV adaptation in *P. infestans*; and (c) investigate whether pathogens with high UV tolerance cause less disease on plants. Knowledge from this study will be important in understanding the potential responses of life particular plant pathogens to future UV radiation and how these responses may impact on the development, distribution, and epidemics of plant diseases as well as approaches suitable for sustainable disease management in agricultural systems.

## 2 MATERIALS AND METHODS

### 2.1 *Phytophthora infestans* collections

Potato leaves infected with *P. infestans* were sampled from seven altitudes located in the Fujian (two sites), Gansu, Guangxi, Guizhou, Ningxia, and Yunnan provinces of China (Table 1) during the 2010 and 2011 growing seasons (Yang et al., 2016). Gansu, Guizhou, Ningxia, and Yunnan are the four top potato production areas in China, while Guangxi and Fujian (Fuzhou and Xiapu) are the two provinces with the highest potential to develop a significant potato industry in coming decades. For all collections, infected leaves were sampled at random from plants separated by 1–2 m and transported to the laboratory within 24 hr for isolation. To isolate the pathogen, infected leaves were first rinsed with running water for 60 s and then with sterilized distilled water for 30 s. A piece of diseased tissue was cut from the margin of a leaf lesion and placed abaxial side up on 2.0% water agar for 20–30 hr. From the resultant sporulating lesion, a single piece of mycelium was removed aseptically using an inoculating needle, transferred to a rye B agar plate supplemented with ampicillin (100 μg/ml) and rifampin (10 μg/ml) and maintained in the dark at 19°C for 7 days to allow a colony to develop. Purification was performed by two sequential transfers of a single piece of mycelium tipped from the colony to a fresh rye B plate. Genotypes of these isolates were previously determined by SSR assay of nuclear genomes (Knapova & Gisi, 2002; Lees et al., 2006), restriction enzyme–PCR amplification of mitochondrial haplotypes (Flier et al., 2003), mating type (Zhu et al., 2015), and partial sequence analysis of three genes (b‐tubulin, Cox1, and Avr3a) (Cárdenas et al., 2011). A total of 140 distinct genotypes, with 20 from each of the seven field populations, were selected for the study. The details of pathogen isolation and molecular characterization of these populations can be found in our previous publications (Qin et al., 2016).

**Table 1:** Geographic coordinates, altitude, and sample size for the seven *Phytophthora infestans* populations were collected

POP	Longitude	Latitude	Altitude (m)	Sample size
Fuzhou	119°17'	26°05'	10	20
Gansu	105°43'	34°35'	2,089	20
Guangxi	108°22'	22°50'	78	20
Guizhou	105°56'	26°16'	1,330	20
Xiapu	119°59'	26°54'	31	20
Ningxia	106°14'	36°01'	1,778	20
Yunnan	102°43'	25°03'	2,677	20

### 2.2 Experimental test for UV tolerance in the *Phytophthora infestans* isolates

*Phytophthora infestans* isolates from long‐term storage were revived on rye B agar at 19℃ for 10 days and then exposed to UV radiation of 10‐, 15‐, 30‐, 90‐, 180‐, 300‐, and 480‐s duration using an ultraviolet light C lamp (PHILIPS, wavelength = 254 nm, 30 w) placed 50 cm above the surface of the colony. Preliminary experiments indicated that these UV doses yielded the best result in differentiating growth rate among the isolates. Many isolates did not grow when higher UV doses were used.

After radiation, mycelial plugs (*ϕ* = 5 mm) were taken from the margin of exposed colonies and transferred to fresh rye B plates supplemented with coccinellin in a 9‐cm Petri dish. Inoculated plots were laid out in a completely randomized design using three replicates (plates) for each isolate in each UV treatment scheme and kept in growth chambers at 19°C for 8 days. A control plate inoculated with a mycelial plug but which was not exposed to UV light was also included in the study for each isolate in each UV scheme. Resultant colonies were photographed each day at 3–8 days after inoculation. Colony sizes were measured with the image analysis software Assess (Lamari, 2002). In total, 3,920 plates (140 isolates × 4 [3 exposed + 1 control] plates × 7 UV irradiation duration periods) were included in the estimate of growth rate. Because of the size of the study, the experiment was conducted in seven stages each corresponding to one of seven UV radiation periods.

### 2.3 Aggressiveness test

Aggressiveness was measured as the area under the disease–progress curve (AUDPC; Fry, 1978) calculated from the 2nd to 5th day after inoculation on Favorite, a cultivar universally susceptible to *P. infestans*, using a detached leaflet approach (Foolad, Sullenberger, & Ashrafi, 2015). Fully expanded leaves excised from Favorite plants grown in the experimental field for 8 weeks were placed on 2% water agar in Petri dishes and then inoculated on the abaxial side with mycelial plugs (*ϕ* = 5 mm) of the 140 isolates. Each isolate was inoculated onto three detached leaves with mycelial plugs. The Petri dishes with detached leaves were arranged in randomized complete block design and maintained at 19℃ in an incubator supplemented with 16‐hr light daily. Leaf and lesion areas were photographed at the 2nd to 5th days post‐inoculation and estimated electronically with the image analysis software Assess (Lamari, 2002).

### 2.4 Data analysis

The UV tolerance of *P. infestans *isolates was estimated by determination of the relative growth rate of colonies in UV‐treated and control (UV untreated) plates. The growth rate of each isolate was estimated using a logistic model (Aguayo, Elegbede, Husson, Saintonge, & Marçais, 2014) based on the sizes of individual colonies quantified at each time point over the 8‐day post‐inoculation period. The initial colony size at the point of inoculation (day one) was set as 0.2 cm^2^ (πr^2^ = 3.14 × 0.25^2^), and the capacity of colony sizes was set to 63.59 cm^2^ (πr^2^ = 3.14 × 4.50^2^). AUDPC was calculated on the basis of disease severity using the trapezoid integration of disease–progress curve over time according to the following formula:

(1) $$\text{AUDPC}=\sum_{i=1}^{n}\left(\frac{x_{i+1}+x_{i}}{2}\right)\left[t_{i+1}-t_{i}\right]$$

where *x_i_*_+1_ and *x_i_* are disease severity at time *t_i_*_+1_ and *t_i_*, and *n* is the total number of observations, respectively. Frequency distribution of relative growth rate in the combined population (pooling all isolates from the locations together) was visualized by grouping isolates into 11 bins differing by 0.07 units. Duncan's multiple range test (Ott, 1992) was used to compare the relative growth rate of *P. infestans* populations sampled from different locations.

Genetic variation and population differentiation in SSR marker loci for the isolates sampled from the seven different locations were taken from a previous publication (Wu et al., 2016). In this analysis, population differentiation for the 8 SSR marker loci was estimated by the fixation index *F*_ST_ (Meirmans & Hedrick, 2011) using POPGENE1.32 (https://sites.ualberta.ca/~fyeh/popgene_download.html). Genetic variation in SSR marker loci was quantified by gene diversity (Nei, 1978). Variances in relative growth rate were calculated and partitioned into sources attribute to isolates (*I*, random effect), population (*P*, random effect) and UV radiation (*T*, fixed effect) using SAS GLM and VARCOMP programs (SAS 9.3 Institute) according to the model:

(2) $$Y_{\text{ripu}}=M+I\left(P\right)+U+P+I\left(P\right)*U+P*U+E_{\text{ripu}}$$

where *Y*_ripu_ is the mean growth rate of replicate *r* for isolate *i* from population *p* at UV radiation dose u; *M* is the overall mean; *U* is the experimental ultraviolet; and *E*_ripu_ is the variance among replicates. The terms *P*, *I* (*P*), *I* (*P*) × *U*, and *P* × *U* refer to genetic variance among populations, genetic variance within populations, variance due to the genotype × ultraviolet radiation interaction, and different responses of populations to changing UV radiation, respectively.

Population differentiation for relative growth rate (*Q*_ST_) was estimated separately for each experimental temperature using a previously described formula (Zhan & McDonald, 2011).

(3) $$Q_{\text{ST}}=\frac{\delta_{\text{AP}}^{2}+\delta_{\text{P}\text{.E}}^{2}/n}{\delta_{\text{AP}}^{2}+\delta_{\text{P}\text{.E}}^{2}/n+\delta_{\text{WP}}^{2}}$$

where $\delta_{\text{AP}}^{2}$, $\delta_{\text{P}\text{.E}}^{2}$, $\delta_{\text{wp}}^{2}$, and *n* are the additive genetic variation attributed to among‐population variation, the variance in population–environment (ultraviolet radiation) interaction, the additive genetic variation attributed to within‐population variation, and the number of environments (ultraviolet radiation schemes), respectively. Heritability of relative growth rate in a population was estimated by dividing genetic variance within populations with total phenotypic variance; phenotypic plasticity of relative growth rate was calculated by dividing the variance of isolate–UV interaction by total phenotypic variance (Tonsor, Elnaccash, & Scheiner, 2013). Statistical differences between the overall *F*_ST_ in SSR loci and overall *Q*_ST_ in UV tolerance were evaluated using the standard deviation of *Q*_ST_ constructed from 100 resampling of the original data (Zhan & McDonald, 2011). Geographic data of seven collection sites were taken from Google Earth©. The associations among parameters such as altitude of collection sites, UV tolerance, UV radiation times, and population differentiation index were evaluated by simple linear correlation (Lin, 1989) or second‐order polynomial correlation (Kniskern & Rausher, 2007).

## 3 RESULTS

### 3.1 Frequency distribution of UV tolerance in field populations of *Phytophthora infestans*

**Figure 1:**
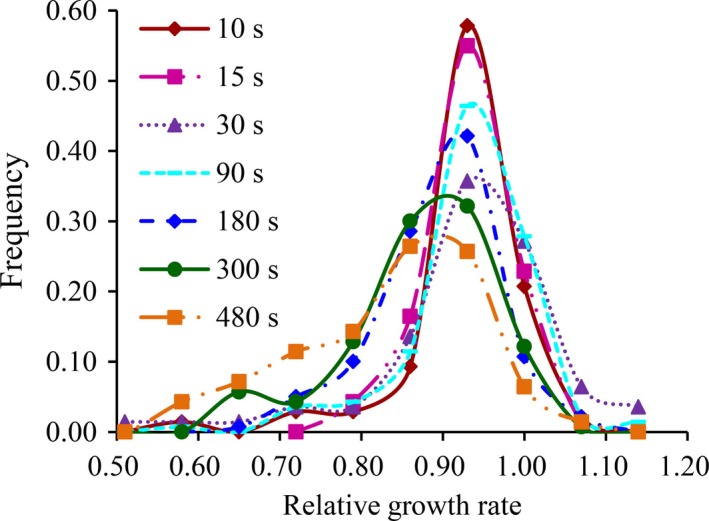
Frequency distribution of tolerance to different UV irradiation in the *Phytophthora infestans* isolates sampled from seven geographic sites varying in altitude

**Figure 2:**
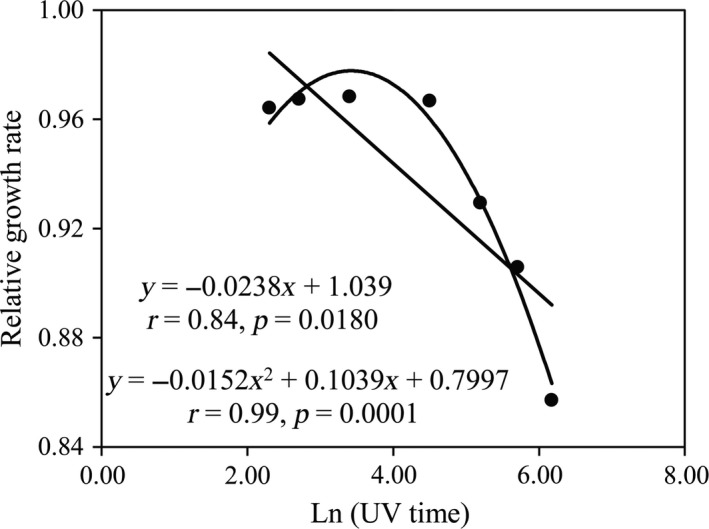
Tolerance (survival) curve of *Phytophthora infestans* isolates in response to the change of experimental UV irradiation

Twenty clonal lineages sampled from each of seven locations across China were tested for UV tolerance by calculating the relative growth rate of the pathogen in the presence and absence of UV treatments. In all seven UV radiation schemes, relative growth rate displayed a continuous and unimodal distribution (Figure 1), indicating a quantitative inheritance of UV tolerance. As UV doses increased, the mode of histograms decreased, leading to a flatter and wider spread of the frequency distribution. The mean relative growth rate of *P. infestans *isolates did not change from 10‐ to 30‐s radiation treatments but decreased quickly as the radiation time increased further (Figure 2). Association analysis revealed that the impact of experimental UV‐C dose on relative growth rate of the pathogen fitted better to a second‐order polynomial (*p* = 0.0001) than a linear relationship (*p* = 0.018) though both models were statistically supported and could be used.

### 3.2 Difference in UV tolerance among *Phytophthora infestans* populations and its association with geographic location

**Figure 3:**
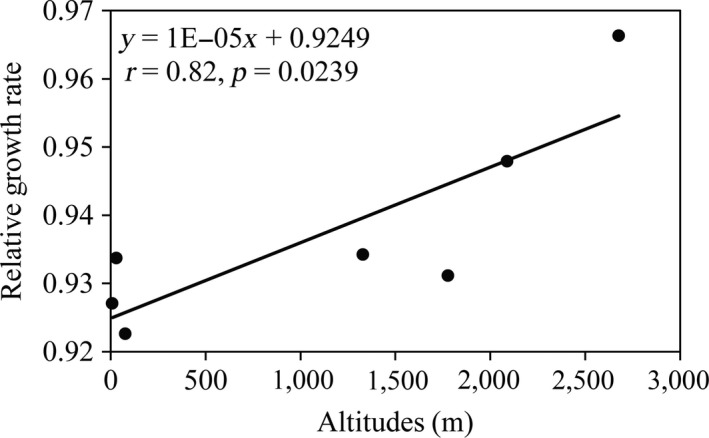
Association between UV tolerances of *Phytophthora infestans* averaged across the seven experimental irradiations and local altitude at collection sites of the pathogen populations

Analysis of variance by general linear model (GLM) indicated population, genotypes, and UV radiation times all contributed significantly (*p* < 0.0001) to differences in UV tolerance among *P. infestans* isolates sampled from different locations. The isolates also responded differentially to seven UV radiation doses as indicated by the significant radiation–genotype interaction (*p* < 0.0001, Table 2). The average relative growth rate across the seven UV radiation schemes ranged from 0.922 to 0.966 with a grand mean of 0.937. With a mean relative growth rate of 0.966, the *P. infestans* population sampled from Yunnan, the highest altitude among the seven locations, displayed the highest tolerance to UV radiation while the pathogen population from Guangxi was the least tolerant (Table 3). Further analysis indicated that the average UV tolerance across the treatment of a *P. infestans* population was significantly and positively correlated with the altitude from which it was sampled (Figure 3). The similar pattern of association was also found when tolerance from individual UV treatments was used (data not shown).

**Table 2:** Analysis of variance (ANOVA) for adaptation to UV irradiation among the *Phytophthora infestans* populations sampled from seven locations in China

Source	*df*	Sum of squares	Mean square	*F* value	*p*
Population	6	0.465	0.077	26.12	<0.0001
Isolates	133	6.455	0.049	16.37	<0.0001
UV	6	5.172	0.862	290.79	<0.0001
UV × isolates	825	28.263	0.0343	11.56	<0.0001
Model	970	140.354	0.042	14.03	<0.0001
Error	1,917	5.682	0.003		

**Table 3:** Genetic diversity of SSR marker loci, mean relative growth rate (a measure of aggressiveness), and disease heritability and plasticity of UV adaptation in 140 *Phytophthora infestans* isolates sampled from seven locations in China

Pop	SSR diversity	AUDPC	UV tolerance			
		Mean	Mean	Heritability	Plasticity	Ratio
Fuzhou	0.44	0.218C	0.927CD	0.061	0.652	10.69
Gansu	0.46	0.178CD	0.937B	0.103	0.691	6.71
Guangxi	0.43	0.363A	0.922D	0.104	0.642	6.17
Guizhou	0.40	0.148D	0.938B	0.012	0.711	59.25
Xiapu	0.49	0.340AB	0.937B	0.044	0.642	14.59
Ningxia	0.39	0.307B	0.930C	0.085	0.699	8.22
Yunnan	0.47	0.168CD	0.966A	0.099	0.650	6.57
Pooled	0.50	0.246	0.937	0.069	0.676	16.03

Note

Values followed by different letters are significantly different from each other at *p* = 0.05. AUDPC: area under the disease–progress curve; SSR: simple sequence repeat.

### 3.3 Genetic diversity in SSR markers and UV tolerance

The SSR diversity across the eight SSR marker loci in the seven *P. infestans* populations ranged from 0.39 to 0.49 with a total of 0.50 (Table 3). The *P. infestans* population sampled from Xiapu displayed the highest level of SSR diversity of 0.49 while that from Ningxia displayed the lowest level of 0.39.

Genetic variance (heritability) accounted for 0.012–0.104 of the phenotypic variance in UV tolerance with a mean of 0.069 (Table 3) while the variance of the isolate–environment interaction (plasticity) accounted for 0.642–0.711 of the phenotypic variation with a mean of 0.676 (Table 2). Plasticity was 6.17‐ to 59.25‐fold (average 16.03) higher than heritability. Gene diversity in SSR marker loci was not correlated with either heritability or plasticity of relative growth rates (data not shown).

### 3.4 Population differentiation in SSR markers and UV tolerance under different radiation times

**Figure 4:**
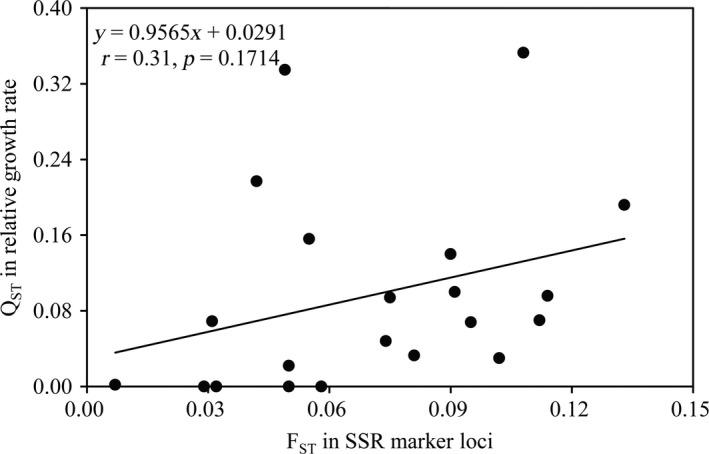
Pairwise correlations between genetic differentiation in the UV tolerance (*Q*_ST_) and simple sequence repeat (SSR) marker loci (*F*_ST_) among the *Phytophthora infestans *populations

The pairwise population differentiation (*F*_ST_) in SSR markers ranged from 0.007 to 0.133, and the pairwise population differentiation (*Q*_ST_) in UV tolerance ranged from 0.000 to 0.353 when all seven radiation schemes were considered together. There was no correlation between pairwise population differentiations in UV tolerance (Figure 4). The overall population differentiation (*Q*_ST_) in UV tolerance when the seven radiation schemes were considered together was 0.141, which was significantly higher than 0.117, the overall population differentiation (*F*_ST_) as measured using SSR markers.

### 3.5 Association of UV tolerance and aggressiveness of *Phytophthora infestans*

**Figure 5:**
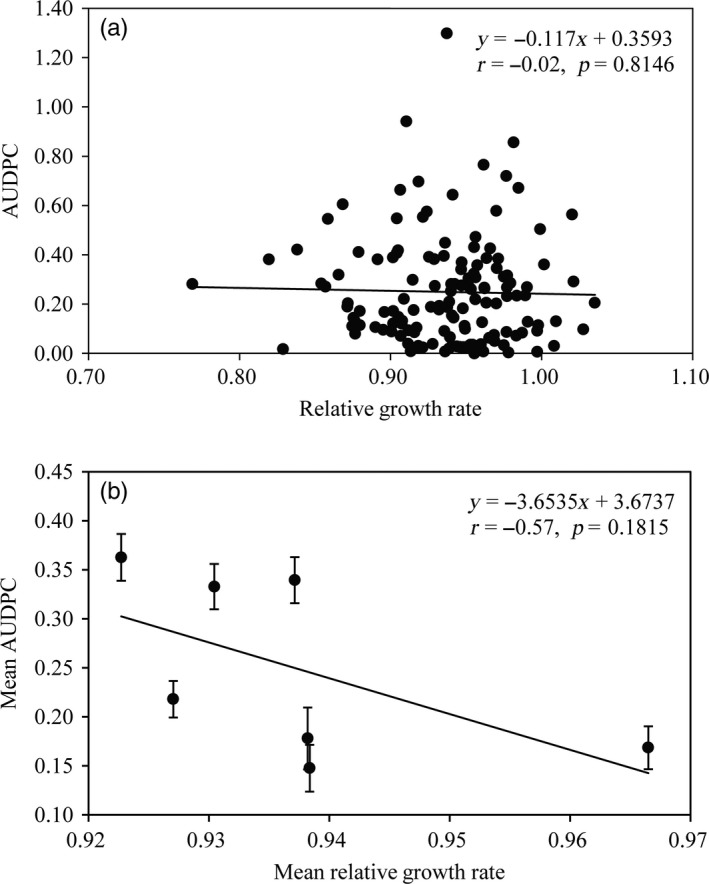
Associations between UV tolerance and aggressiveness measured by percentage of leaf area covered by lesion in the *Phytophthora infestans *isolates: (a) individual isolate and (b) population mean

There were significant differences in aggressiveness (as measured by the AUDPC) among populations and isolates of *P. infestans *(*p* < 0.001). Average aggressiveness in the seven field populations ranged from 0.148 to 0.363 with a grand mean of 0.246 (Table 3). The pathogen population originated from Guangxi displayed the highest aggressiveness while that from Guizhou had the lowest aggressiveness. Aggressiveness and UV tolerance of *P. infestans* were not correlated at either the isolate (Figure 5a, *r* = −0.02, *p* = 0.8146) or population (Figure 5b, *r* = −0.57, *p* = 0.1815) levels.

## 4 DISCUSSION

Climate change, a multiple‐component phenomenon involving long‐term shifts in the average and distribution of many weather parameters such as air temperature, UV radiation, and precipitation (Häder et al., 2015; Milchunas et al., 2004; Siepielski et al., 2017; Zhan, Ericson, & Burdon, 2018), is posing an imminent threat to the earth. Intergovernmental Panel on Climate Change (IPCC) and other scientific assessments predict that the current cycle of climate change may continue for decades (Altizer et al., 2013; Gleick et al., 2010; IPCC, 2014) and warn that immediate actions should be taken to mitigate potentially and irreversibly catastrophic impacts of climate change on ecological functions and sustainability. As a part of ongoing climate change, UV radiation is expected to increase in the next decades as a consequence of stratospheric ozone depletion caused by increasing emission of industrial gases (Petkov et al., 2014). Great concern has been raised by scientists and public on how organisms on the earth may respond to the increase in UV radiation biologically, genetically, and evolutionarily and how these responses may influence function and integrity of natural ecosystems, sustainability of food production, and health of human and animals (Häder et al., 2015; Kashian, Zuellig, Mitchell, & Clements, 2007). In this study, we combined physiological growth assays, molecular genetic markers, and a population and quantitative genetic approach to examine the evolutionary adaptation of *P. infestans* to UV radiation by comparing levels of UV tolerance among seven *P. infestans* populations collected at different altitudes. We found significant divergence in UV tolerance among geographic populations (Table 3). Spatial population divergence in ecological traits can be generated by adaptive process in response to natural selection imposed by local biotic and abiotic environments or a non‐adaptive process through genetic drift such as founder events, bottlenecks, and/or the random fixation of alleles (Dowle, Morgan‐Richards, Brescia, & Trewick, 2015). Distinguishing these two evolutionary processes is challenging but can be achieved by comparative analysis of spatial population genetic dynamics in the ecological traits concerned and neutral marker loci (Spitze, 1993; Zhan & McDonald, 2011; Zhan et al., 2005). Diversifying selection in response to local biotic and abiotic environments increases population differentiation for ecological traits (*Q*_ST_), causing a significantly higher *Q*_ST_ than population differentiation in neutral marker loci (*F*_ST_). If spatial population divergence in the ecological traits results from genetic drift, *Q*_ST_ is expected to be similar to *F*_ST_ (Yang, Yeh, & Yanchuk, 1996), leading to positive and linear correlation between the two measurements. When spatial population genetic structures of UV tolerance and SSR marker loci were compared, we found that *Q*_ST_ was significantly higher than *F*_ST_, suggesting that the spatial polymorphism in UV tolerance among *P. infestans* populations is caused by diversifying selection for local adaptation. Natural selection on UV adaptation driven by local environments may also explain the nonlinear correlation between *Q*_ST_ and *F*_ST_ in this study (Figure 4).

A positive correlation was found between UV tolerance and the altitude of *P. infestans *populations, suggesting that the UV tolerance of the pathogen tends to be greater in populations occurring at higher altitudes (Figure 3). No such association was found between UV tolerance and other meteorological parameters such as local temperature, humidity, or rainfall (data not shown). The extent of UV radiation received at any given location on the earth's surface is mainly affected by its distance above sea level and increases ~10% for every 1,000 m increase in altitude (Blumthaler, Ambach, & Ellinger, 1997). Our results support the hypothesis that diversifying selection acting through difference in local UV environments is the primary mechanism driven the evolution of UV adaptation in *P. infestans* populations. This pattern of natural selection and local adaptation is likely to be common in both agricultural and natural ecosystems when considering UV radiation is a potent stressor that can damage DNA structure and interrupt biochemical and biological function of species (Scrima et al., 2008).

Genetic polymorphism is fundamental to the ability of species to cope with environmental stresses such as UV radiation and provides raw material for natural selection. In natural populations, two categories of polymorphism (i.e., additive genetic variance and plasticity) usually coexist in many biologically important traits (Huchard et al., 2014). Additive genetic variance generated by changes in DNA sequences results in permanent adaptation of species to local environments, while plasticity, which is also inheritable (Pelletier, Réale, Garant, Coltman, & Festa‐Bianchet, 2007), is a phenomenon whereby a genotype produces different phenotypes through modifying gene expression and/or enzymatic activity in response to environmental fluctuation (Draghi & Whitlock, 2012). The relative importance of the two polymorphisms in the evolution of species is both trait‐ and environment‐dependent (He et al., 2018; Qin et al., 2016; Yang et al., 2016). It is predicted that the importance of plasticity relative to additive genetic variance increases in populations originating from geographic locations experiencing greater environment fluctuation and to be less important in environments that are more stable (Draghi & Whitlock, 2012). In the current study, plasticity contributed ~16‐fold higher variance than additive genetic variance to the natural variation in UV tolerance, suggesting temporary modification in gene expression and/or enzymatic activity played a more important role than permanent changes in gene structure in the evolution of UV adaptation. Changes in gene expression and/or enzymatic activity can be induced on a shorter timescale than functional changes in DNA sequences and are reversible, minimizing the lag‐time in adaptation to environmental stresses and the impact of any potentially long‐term fitness penalty associated with permanent change of gene structure through mutation. Given the constant and dramatic change of UV radiation across seasons or sometimes within minutes in the same habitats as a result of tidal cycles and weather conditions (Ballaré, Caldwell, Flint, Robinson, & Bornman, 2011; Magri, 2011), such an adaptive mechanism is expected to be selectively favored and have lower fitness costs (Chevin, Lande, & Mace, 2010). In addition, plasticity might fit better to pathogens with the ability of long‐distance dispersal such as *P. infestans *(due to frequent movement of propagules among altitudes).

Indeed, no negative association was detected between UV tolerance and aggressiveness of the pathogen when a linear correlation analysis was conducted (Figure 5), suggesting low or no fitness penalty to genotypes with higher UV tolerance. This result is consistent with some previous reports from other systems (Miner & Kerr, 2011). Though other explanations cannot be completely excluded, the adaptive strategies developed by the pathogen are likely to be the primary contribution to the current observation. The majority of population variation in UV tolerance is generated by plasticity through the temporary regulation of gene expression and/or enzymatic activity while the contribution of additive genetic variance by permanent change in gene structure via mutation is negligible (Table 3). As discussed earlier, these adaptive strategies are cost‐effective by minimizing potential negative pleiotropy with other biological processes caused by anti‐ultraviolet mutations interrupting the normal biochemical functions of genes (Bess et al., 2013).

In conclusion, elevation of UV radiation associated with anthropogenic activity can exert critical influences on many aspects of biological, ecological, and evolutionary processes of species (Cable et al., 2017; Roig‐Sagués, Gervilla, Pixner, Terán‐Peñafiel, & Hernández‐Herrero, 2018; Siepielski et al., 2017; Zhan & McDonald, 2011). In agricultural and medical fields, there is considerable concern that such trend may have major impacts on disease ecology. However, a general lack of evolutionary understanding as to how plant pathogens may response to future change of UV radiation prevents confident prediction. Our analysis of the evolutionary mechanisms, processes, and trade‐off involved in UV adaptation will hopefully stimulate further comprehensive studies in this field.

## CONFLICT OF INTEREST

None declared.

## Data Availability

Data available from the Dryad Digital Repository: https://doi.org/10.5061/dryad.t0f998t.
